# Supplementary material for: Pulsed stimuli enable p53 phase resetting to synchronize single cells and modulate cell fate
Source: Mol Syst Biol. 2025 Mar 3;21(4):390–412. doi: 10.1038/s44320-025-00091-8 (PMC11965341; doi:10.1038/s44320-025-00091-8)
Supplement: Supplementary file 1 — Table EV1 [file 44320_2025_91_MOESM1_ESM.docx]

**Table EV1. Parameter symbols and descriptions.** A list of the parameter symbols, descriptions, and values used in the computational modeling.

| **Symbol** | **Parameter description** | **Value** |
| --- | --- | --- |
| A | ATM autophosphorylation rate constant | 30.5 |
| P | Wip1-dependent ATM dephosphorylation rate constant | 22 |
| C | Basal p53 synthesis rate constant | 1.4 |
| g | Mdm2-dependent p53 degradation rate constant | 2.5 |
| d_AM_ | ATM-dependent Mdm2 degradation rate constant | 20 |
| T_mdm2_ | MDM2 mRNA production rate constant | 1.2 |
| T_M_ | Mdm2 protein production rate constant | 4 |
| T_wip1_ | WIP1 mRNA production rate constant | 1.2 |
| T_W_ | Wip1 protein production rate constant | 1 |
| d_A_ | Basal ATM dephosphorylation rate constant | 0.16 |
| d_P_ | Basal p53 degradation rate constant | 0.1 |
| d_mdm2_ | MDM2 mRNA degradation rate constant | 1 |
| d_M_ | Mdm2 protein degradation rate constant | 2 |
| d_wip1_ | WIP1 mRNA degradation rate constant | 1.3 |
| d_W_ | Wip1 protein degradation rate constant | 2.3 |
| k_A_ | Michaelis constant for ATM autophosphorylation | 0.5 |
| k_WA_ | Michaelis constant for Wip1-dependent ATM dephosphorylation | 0.14 |
| k_MP_ | Michaelis constant for Mdm2-dependent p53 degradation | 0.15 |
| k_Pm_ | Michaelis constant for p53-dependent MDM2 mRNA production | 1 |
| k_Pw_ | Michaelis constant for p53-dependent WIP1 mRNA production | 1 |
| R | Strength of inhibition of Mdm2-dependent p53 degradation by ATM | 2 |
| S_max_ | Strength of ATM activation by DSBs | 0.2 |
| gamma | Michaelis constant of ATM activation by DSBs | 9 |
