# Supplementary material for: Pulsed stimuli enable p53 phase resetting to synchronize single cells and modulate cell fate
Source: Mol Syst Biol. 2025 Mar 3;21(4):390–412. doi: 10.1038/s44320-025-00091-8 (PMC11965341; doi:10.1038/s44320-025-00091-8)
Supplement: Supplementary file 2 — Table EV2 [file 44320_2025_91_MOESM2_ESM.docx]

**Table EV2. Exact p-values for statistical tests.** A list of exact p-values for all statistical tests performed.

| **Figure** | **Comparison** | **Statistical test** | **p-value** |
| --- | --- | --- | --- |
| 1E | 1^st^ peak timing | Kolmogorov Smirnov test | 0.9323 |
|  | 2^nd^ peak timing | Kolmogorov Smirnov test | 0.0017 |
| 1F | Single Dose vs. 5.5 | See 2B | See 2B |
| 2B | \| Single Dose vs. 3 \| \| --- \| \| Single Dose vs. 3.5 \| \| Single Dose vs. 4 \| \| Single Dose vs. 4.5 \| \| Single Dose vs. 5 \| \| Single Dose vs. 5.5 \| \| Single Dose vs. 6 \| \| Single Dose vs. 6.5 \| \| Single Dose vs. 7 \| | ANOVA followed by multiple comparisons | \| 0.4261 \| \| --- \| \| 0.7139 \| \| 0.0040 \| \| 0.0077 \| \| 0.0059 \| \| 0.0240 \| \| 0.4214 \| \| 0.1958 \| \| 0.9420 \| |
| 4C | 1^st^ peak timing | Wilcoxon rank sum test | 0.6986 |
|  | 2^nd^ peak timing | Wilcoxon rank sum test | <0.0001 (7.7916e-16) |
| 4G | 4.0 h vs 5.5 h | t-test on log-fold change | 0.0128 |
| 5B GADD45A | Overall | ANOVA (treatment factor) | 0.0337 |
|  | \| 5h \| \| --- \| \| 6h \| \| 7h \| \| 8h \| | Post-hoc corrected for multiple comparisons | \| 0.0801 \| \| --- \| \| 0.0088 \| \| 0.0584 \| \| 0.9998 \| |
| 5B  CDKN1A | Overall | ANOVA (treatment factor) | 0.3484 |
|  | \| 5h \| \| --- \| \| 6h \| \| 7h \| \| 8h \| | Post-hoc corrected for multiple comparisons | \| 0.7052 \| \| --- \| \| 0.8484 \| \| 0.5058 \| \| >0.9999 \| |
| 5B  FAS | Overall | ANOVA (treatment factor) | 0.2189 |
|  | \| 5h \| \| --- \| \| 6h \| \| 7h \| \| 8h \| | Post-hoc corrected for multiple comparisons | \| >0.9999 \| \| --- \| \| >0.9999 \| \| 0.0716 \| \| 0.3449 \| |
| 5B  TRIAP1 | Overall | ANOVA (treatment factor) | 0.1759 |
|  | \| 5h \| \| --- \| \| 6h \| \| 7h \| \| 8h \| | Post-hoc corrected for multiple comparisons | \| 0.7063 \| \| --- \| \| 0.9995 \| \| 0.5280 \| \| 0.9557 \| |
| 5D  BTG2 | Overall | ANOVA (treatment factor) | 0.0014 |
|  | \| 6h \| \| --- \| \| 7h \| \| 8h \| | Post-hoc corrected for multiple comparisons | \| \| 0.1626 \| \| --- \| \| 0.1465 \| \| 0.3027 \| \| \| --- \| --- \| --- \| --- \| |
| 5D  PMAIP1 | Overall | ANOVA (treatment factor) | 0.0400 |
|  | \| 6h \| \| --- \| \| 7h \| \| 8h \| | Post-hoc corrected for multiple comparisons | \| 0.0371 \| \| --- \| \| 0.0762 \| \| 0.7196 \| |
| 5D  PUMA | Overall | ANOVA (treatment factor) | 0.0510 |
|  | \| 6h \| \| --- \| \| 7h \| \| 8h \| | Post-hoc corrected for multiple comparisons | \| 0.3764 \| \| --- \| \| 0.7072 \| \| 0.1979 \| |

| **Figure** | **Comparison** | **Statistical test** | **p-value** |
| --- | --- | --- | --- |
| EV2B | 1^st^ IPI | p-value of non-zero slope in linear model | 0.010222 |
|  | 2^nd^ IPI | p-value of non-zero slope in linear model | 0.16848 |
| EV4D | See 4G | See 4G | See 4G |
| EV5B  GADD45A | Overall | ANOVA (treatment factor) | <0.0001 |
|  | \| 5h \| \| --- \| \| 6h \| \| 7h \| \| 8h \| | Post-hoc corrected for multiple comparisons | \| 0.0288 \| \| --- \| \| 0.0014 \| \| 0.3075 \| \| 0.7338 \| |
| EV5B  CDKN1A | Overall | ANOVA (treatment factor) | 0.1732 |
|  | \| 5h \| \| --- \| \| 6h \| \| 7h \| \| 8h \| | Post-hoc corrected for multiple comparisons | \| 0.2446 \| \| --- \| \| 0.9557 \| \| 0.9971 \| \| 0.9701 \| |
| EV5B  FAS | Overall | ANOVA (treatment factor) | 0.0812 |
|  | \| 5h \| \| --- \| \| 6h \| \| 7h \| \| 8h \| | Post-hoc corrected for multiple comparisons | \| 0.3905 \| \| --- \| \| 0.9201 \| \| 0.0669 \| \| 0.2885 \| |
| EV5B  TRIAP1 | Overall | ANOVA (treatment factor) | 0.1666 |
|  | \| 5h \| \| --- \| \| 6h \| \| 7h \| \| 8h \| | Post-hoc corrected for multiple comparisons | \| 0.9987 \| \| --- \| \| 0.9940 \| \| 0.4627 \| \| 0.6471 \| |
| EV5C GADD45A | Overall | ANOVA (treatment factor) | <0.0001 |
|  | \| 5h \| \| --- \| \| 6h \| \| 7h \| \| 8h \| | Post-hoc corrected for multiple comparisons | \| 0.0463 \| \| --- \| \| <0.0001 \| \| 0.0022 \| \| 0.9997 \| |
| EV5C  CDKN1A | Overall | ANOVA (treatment factor) | 0.1637 |
|  | \| 5h \| \| --- \| \| 6h \| \| 7h \| \| 8h \| | Post-hoc corrected for multiple comparisons | \| 0.6517 \| \| --- \| \| 0.3156 \| \| 0.6017 \| \| 0.5202 \| |
| EV5C  FAS | Overall | ANOVA (treatment factor) | 0.2006 |
|  | \| 5h \| \| --- \| \| 6h \| \| 7h \| \| 8h \| | Post-hoc corrected for multiple comparisons | \| 0.9552 \| \| --- \| \| 0.9901 \| \| 0.4928 \| \| 0.8182 \| |
| EV5C  TRIAP1 | Overall | ANOVA (treatment factor) | <0.0001 |
|  | \| 5h \| \| --- \| \| 6h \| \| 7h \| \| 8h \| | Post-hoc corrected for multiple comparisons | \| 0.6857 \| \| --- \| \| 0.2329 \| \| 0.0024 \| \| 0.0273 \| |
| EV5D  GADD45A | Overall | ANOVA (treatment factor) | 0.0001 |
|  | \| 5h \| \| --- \| \| 6h \| \| 7h \| \| 8h \| | Post-hoc corrected for multiple comparisons | \| 0.0025 \| \| --- \| \| 0.1437 \| \| 0.0029 \| \| 0.9720 \| |
| EV5D  CDKN1A | Overall | ANOVA (treatment factor) | <0.0001 |
|  | \| 5h \| \| --- \| \| 6h \| \| 7h \| \| 8h \| | Post-hoc corrected for multiple comparisons | \| 0.1391 \| \| --- \| \| 0.2288 \| \| <0.0001 \| \| 0.5032 \| |
| EV5D  FAS | Overall | ANOVA (treatment factor) | 0.0001 |
|  | \| 5h \| \| --- \| \| 6h \| \| 7h \| \| 8h \| | Post-hoc corrected for multiple comparisons | \| 0.9558 \| \| --- \| \| >0.9999 \| \| 0.0001 \| \| 0.0057 \| |
| EV5D  TRIAP1 | Overall | ANOVA (treatment factor) | 0.3238 |
|  | \| 5h \| \| --- \| \| 6h \| \| 7h \| \| 8h \| | Post-hoc corrected for multiple comparisons | \| 0.0220 \| \| --- \| \| 0.4581 \| \| 0.5444 \| \| 0.1371 \| |
