# Supplementary material for: Pulsed stimuli enable p53 phase resetting to synchronize single cells and modulate cell fate
Source: Mol Syst Biol. 2025 Mar 3;21(4):390–412. doi: 10.1038/s44320-025-00091-8 (PMC11965341; doi:10.1038/s44320-025-00091-8)
Supplement: Supplementary file 4 — Expanded View Figures [file 44320_2025_91_MOESM4_ESM.pdf]

Expanded View Figures

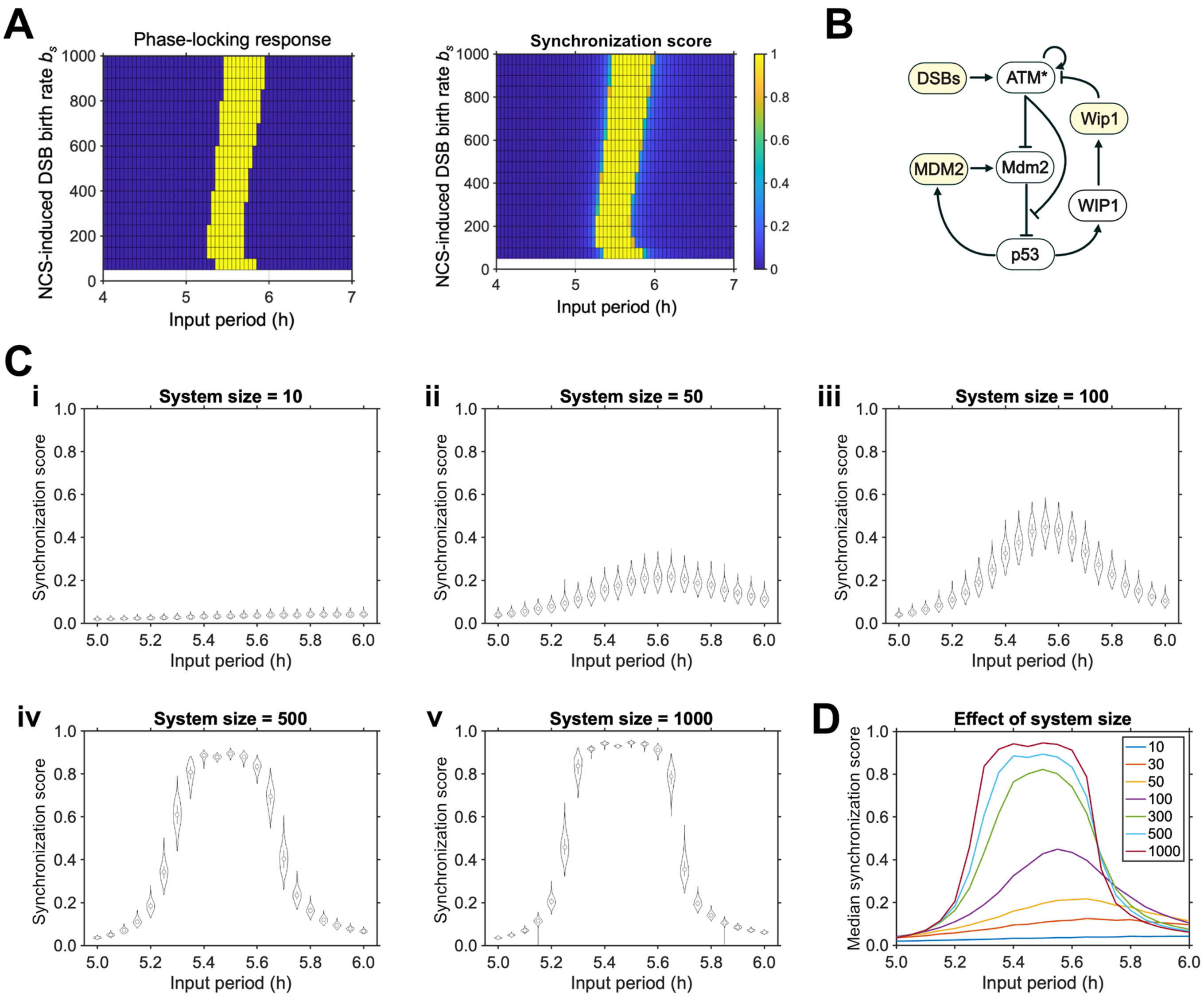

Figure EV1. Synchronization score characterization for the deterministic and stochastic p53 models.

(**A**) Phase-locking response and synchronization scores as a function of NCS-induced birth rate and input period. Yellow regions represent phase-locking while blue represents no phase-locking. (**B**) Diagram of the p53 regulatory network responsive to DSBs. Yellow boxes denote species with stochasticity. (**C**) Violin plots of the top 99% of synchronization scores for $n = 1000$ stochastic simulations of the p53 DSB response in different system sizes at different NCS input periods for an NCS-induced break rate of 200 breaks/h. Here, system size refers to the factor used to convert concentration to number of molecules in the stochastic simulations. Larger system size implies more molecules and, therefore, less noise. Conversely, smaller system size implies fewer molecules and more noise. (**D**) Median synchronization score of 1000 stochastic simulations of the p53 DSB response in different system sizes at different NCS input periods for an NCS-induced break rate of 200 breaks/h. Overall, there is no increase in synchronization range due to intrinsic noise in comparison to the deterministic system.

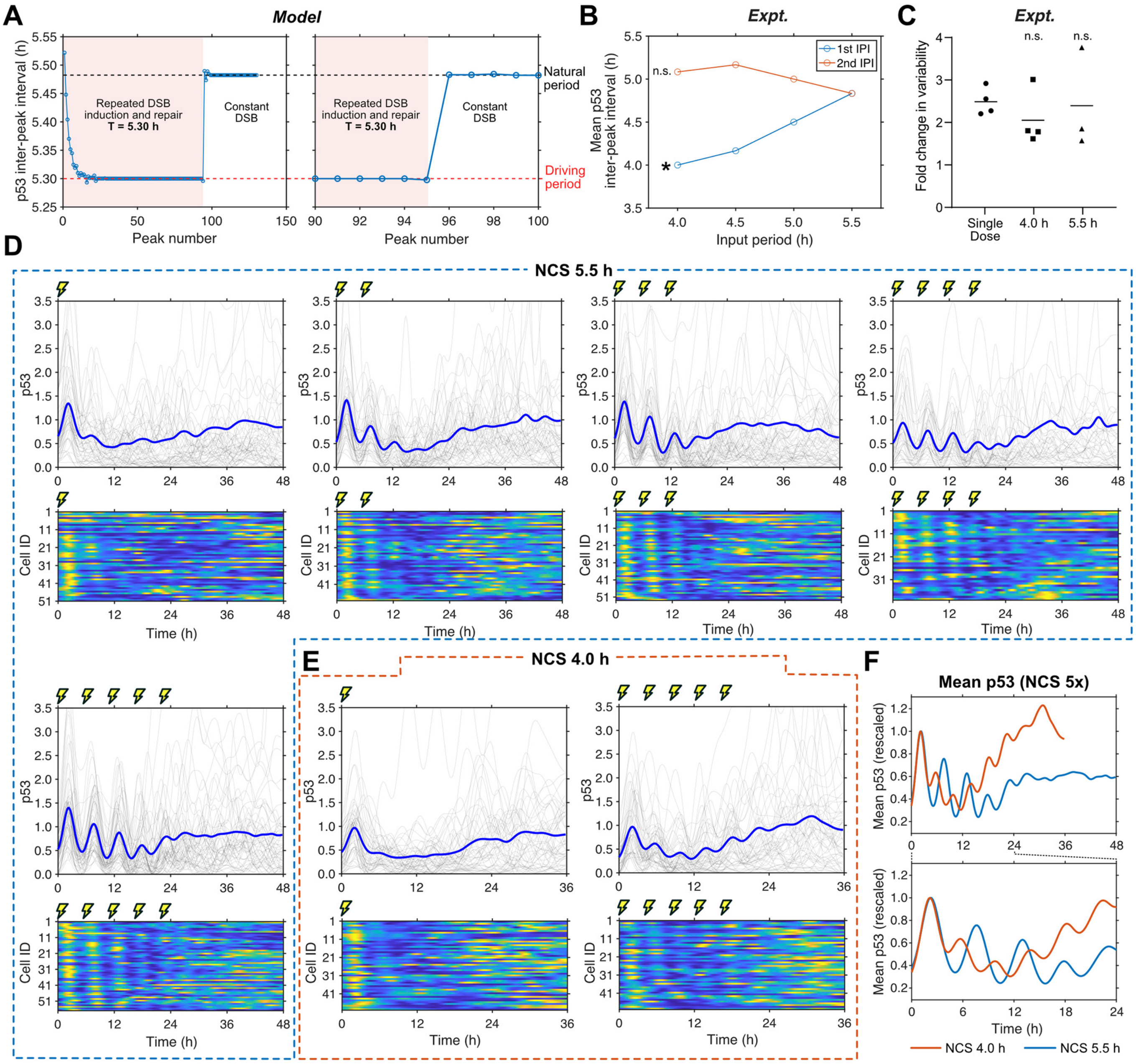

Figure EV2. p53 synchronization is consistent with phase resetting rather than entrainment.

(**A**) Graph of the p53 interpeak interval in response to repeated DSB induction at a period of 5.3 h (red area) and after a shift to constant levels of DSB (white area). (**B**) Mean p53 interpeak interval as a function of the input period quantified from the data in Fig. 2 (**P* < 0.05; *P* value indicates the statistical significance of a non-zero slope in a linear regression vs. input period. Exact *P* values provided in Table EV2). (**C**) Dot plots showing the fold change in peak timing variability when there is no phase-resetting dose. These are the 2nd to 1st peak fold changes for the single-dose case and the 3rd to 2nd peak fold changes for the 4.0 h and 5.5 h cases. ($n = 4$, 4, and 3 biological replicates from left to right; statistical significance calculated using an ANOVA). (**D**, **E**) Single-cell traces (gray) and mean (dark blue) line plots and the corresponding per-cell rescaled heatmaps of p53-mVenus expression under one to five doses of NCS (400 ng/mL) spaced 5.5 h apart (**D**, enclosed in the blue dashed outline) along with those from cells treated with one and five doses of NCS spaced 4.0 h apart (**E**, enclosed in the orange dashed outline). (**F**) Mean p53 expression of cells treated with five doses of NCS (400 ng/mL) spaced 4.0 h (orange) or 5.0 h (blue) apart showcasing the difference in pulse frequencies during the dosing regimens.

 © The Author(s)

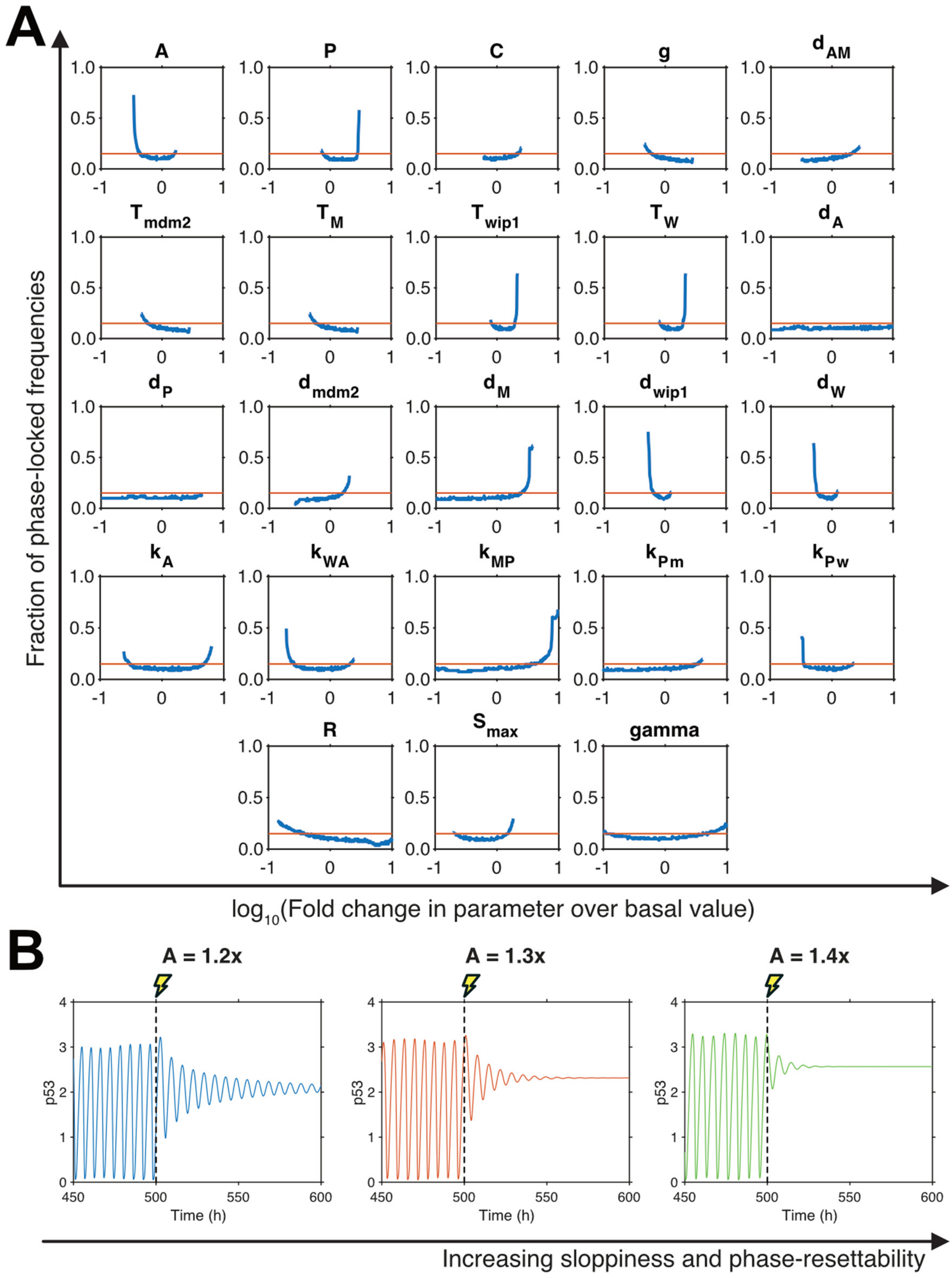

Figure EV3. Effect of system parameters on synchronizability and transition to non-oscillatory regimes.

(**A**) Effect of log_{10} fold change in each parameter on the fraction of input periods in a scaled window around the natural oscillatory period of the system. Effect of parameters considered significant if the y-axis value exceeded 0.15 (orange line, ~50% increase over basal synchronization range). Explanation of symbols provided in Table EV1. (**B**) Change in p53 dynamics due to a sudden and large sustained increase in levels of DNA damage at $t = 500$ h for different values of the ATM autophosphorylation rate constant (parameter A). A higher value of A results in a more phase-resettable system which corresponds to a faster decay in the transient oscillations in reaching the new stable steady state.

© The Author(s)

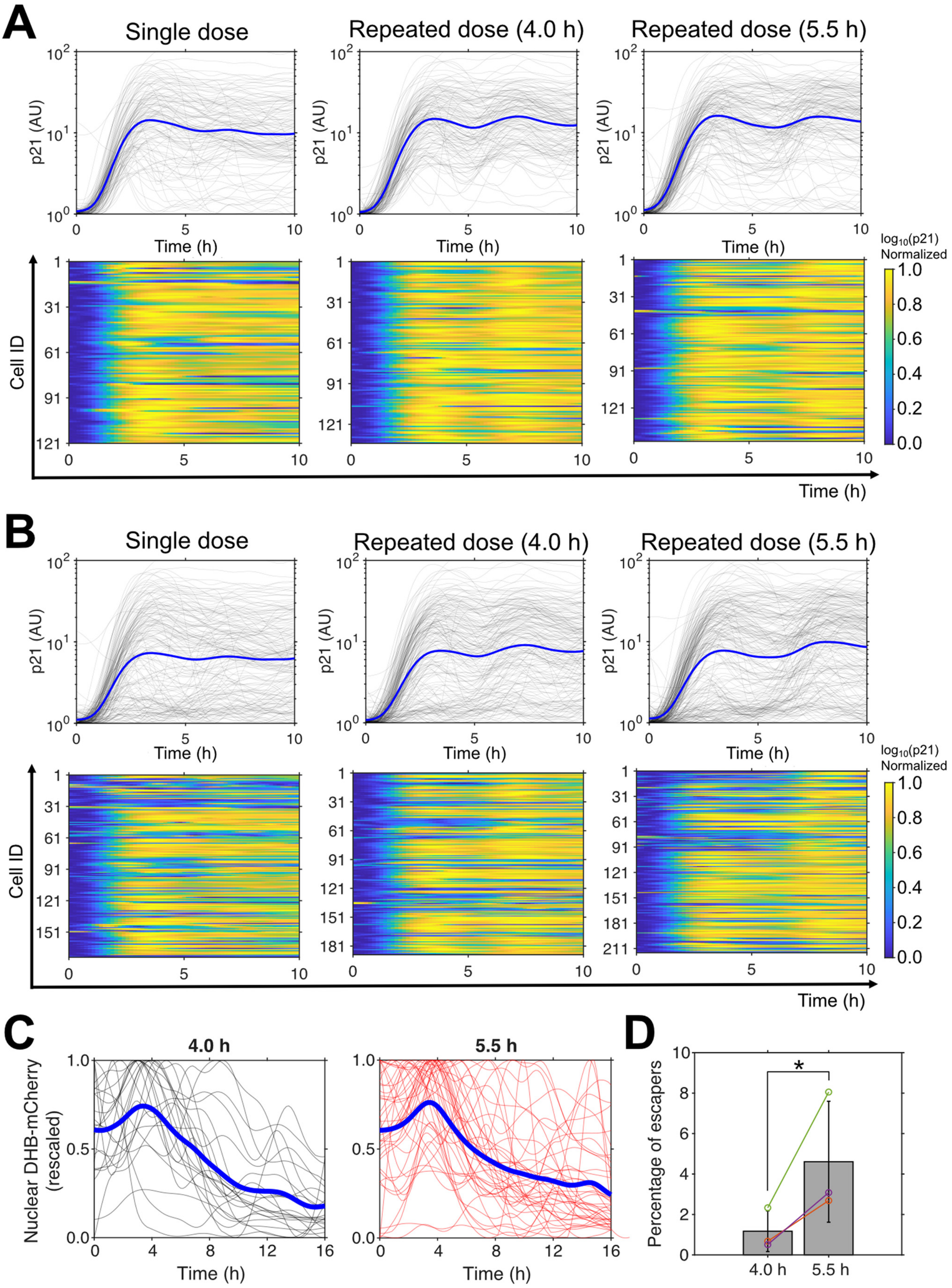
A
Single dose
Repeated dose (4.0 h)
Repeated dose (5.5 h)
p21 (AU)
Time (h)
Cell ID
log10(p21) Normalized
B
Single dose
Repeated dose (4.0 h)
Repeated dose (5.5 h)
p21 (AU)
Time (h)
Cell ID
log10(p21) Normalized
C
4.0 h
5.5 h
Nuclear DHB-mCherry (rescaled)
Time (h)
D
Percentage of escapers
4.0 h
5.5 h

 © The Author(s)

Figure EV4. p21 expression in single cells treated with different NCS dosing regimens and Cdk2 activity in escaper cells.

(**A**) Single-cell traces (gray) and mean p21 expression values (blue) for conditions from Fig. 4 along with a single dose of NCS as well as heat map diagrams for these conditions where each p21 trace is rescaled from 0 to 1. $n = 121$, 135, and 148 for the single dose, double dose (4.0 h) and double dose (5.5 h) conditions, respectively. (**B**) All single-cell traces of p21 expression without filtering for responding cells showing variability in timing of p21 induction despite the same treatment conditions (as reported previously (Sheng et al, 2019)). $n = 175$, 190, and 216 for the single dose, double dose (4.0 h) and double dose (5.5 h) conditions, respectively. (**C**) Nuclear DHB-mCherry fluorescence in escaper cells (rescaled from 0 to 1 on a per-cell basis) for the 4.0 h and 5.5 h 3× repeated NCS treatment regimes. (**D**) Percentage of escaper cells for the 4.0 h and 5.5 h 3× repeated NCS treatment regimes. Bar plots and error bars indicate the mean and standard deviation of three biological replicates. Each line plot corresponds to the percentage of escaper cells from one replicate. Statistical significance calculated by a *t* test on the log fold change with a null hypothesis of log fold change = 0 (*$P < 0.05$. Exact *P* values provided in Table EV2).

© The Author(s)

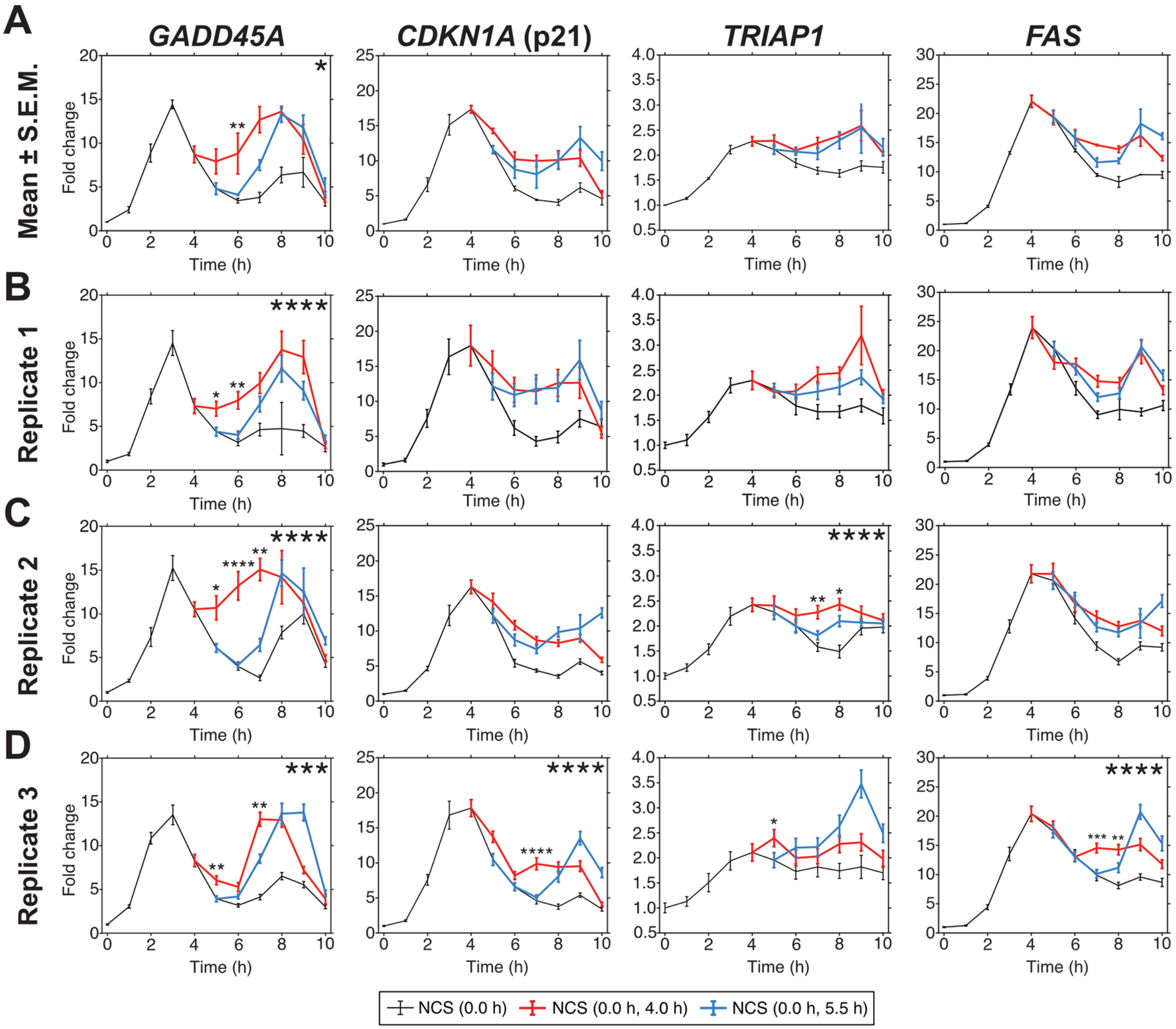

Figure EV5. p53 target gene expression as a function of NCS dose interval.

Gene expression of *GADD45A*, *CDKN1A*, *TRIAP1*, and *FAS* as measured by qRT-PCR in MCF-7 p53-mVenus cells treated with a single dose (black lines) or double dose of NCS at 4.0 h or 5.5 h intervals (red or blue lines, respectively). (**A**) Mean and SEM of three biological replicates. (**B–D**) Mean and standard deviation of the three technical replicates within each biological replicate. Statistical significance of the difference in gene expression between the 4.0 h treatment and 5.5 h treatment from 5.0 h to 8.0 h is shown on the top right of each plot. Statistical significance of the differences in gene expression at a specific time between the two conditions is shown on top of each point. (**P* < 0.05; ***P* < 0.01; ****P* < 0.001; *****P* < 0.0001; repeated measures ANOVA followed by multiple comparisons. The subject factor, not the time factor, of the repeated measures ANOVA is reported here. Exact *P* values provided in Table EV2.)

 © The Author(s)

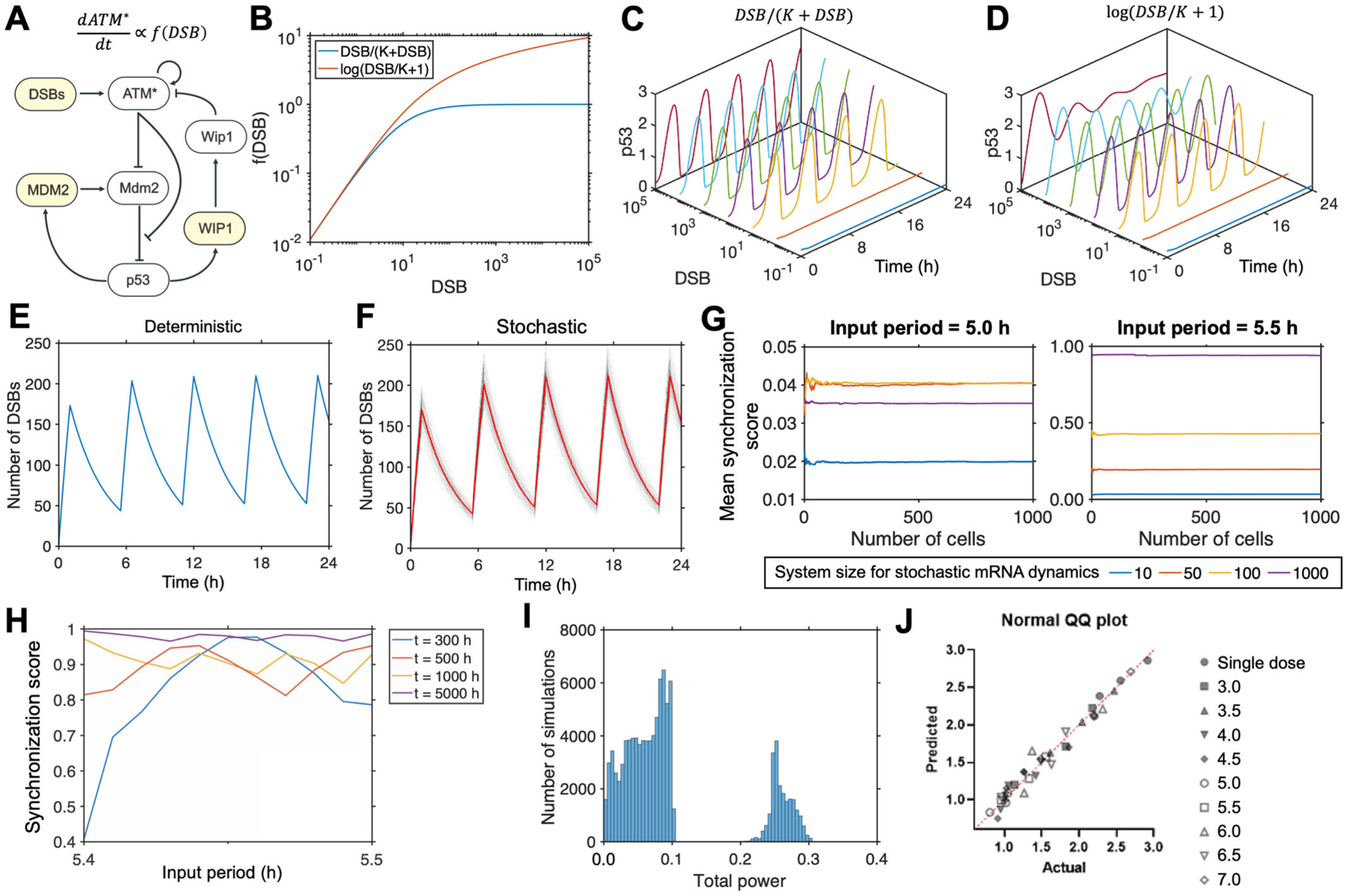

Figure EV6. Modifications to the mathematical model and statistical considerations.

(**A**) Schematic of the p53 DSB response network where the rate of change in ATM* is directly proportional to a DSB input function. (**B**) Plots of the saturable and non-saturable DSB input functions for different values of DSBs. (**C**, **D**) Simulations of the p53 system with the saturable (**C**) and non-saturable (**D**) DSB input functions for different values of DSBs showing similar behavior at low damage but divergent behavior at high damage. (**E**) Deterministic DSB induction and repair with NCS-induced damage being applied every 5.5 h with a NCS-induced birth rate of 200 breaks/h, showing a characteristic sawtooth shape. (**F**) Stochastic DSB inductions and repair with same conditions as (**E**). Gray lines represent individual realizations of the stochastic process; the red line represents the mean across multiple runs. (**G**) Plot showing the average synchronization score as a function of the number of stochastic realizations for a non-entraining and an entraining input period under different levels of noise. Each plot shows convergence well before 1000 instances. (**H**) Synchronization score of the fully deterministic system for different simulation lengths showing fluctuations in shorter simulations due to the finite numerical nature of the DFT used to calculate the synchronization score. (**I**) Histogram of the total spectral power at steady state for systems with parameter sets randomly sampled in a twofold range around the basal values and simulated with 0 to 1000 DSBs, in intervals of 20, as the input. (**J**) QQ plot of the Shapiro–Wilk normality test for data in Figs. 1 and 2.
